# Preoperative Applications of Navigated Transcranial Magnetic Stimulation

**DOI:** 10.3389/fneur.2020.628903

**Published:** 2021-01-22

**Authors:** Alexander F. Haddad, Jacob S. Young, Mitchel S. Berger, Phiroz E. Tarapore

**Affiliations:** ^1^School of Medicine, University of California, San Francisco, San Francisco, CA, United States; ^2^Department of Neurological Surgery, University of California, San Francisco, San Francisco, CA, United States

**Keywords:** TMS, transcranial magnetic stimulation, motor mapping, language mapping, preoperative

## Abstract

Preoperative mapping of cortical structures prior to neurosurgical intervention can provide a roadmap of the brain with which neurosurgeons can navigate critical cortical structures. In patients undergoing surgery for brain tumors, preoperative mapping allows for improved operative planning, patient risk stratification, and personalized preoperative patient counseling. Navigated transcranial magnetic stimulation (nTMS) is one modality that allows for highly accurate, image-guided, non-invasive stimulation of the brain, thus allowing for differentiation between eloquent and non-eloquent cortical regions. Motor mapping is the best validated application of nTMS, yielding reliable maps with an accuracy similar to intraoperative cortical mapping. Language mapping is also commonly performed, although nTMS language maps are not as highly concordant with direct intraoperative cortical stimulation maps as nTMS motor maps. Additionally, nTMS has been used to localize cortical regions involved in other functions such as facial recognition, calculation, higher-order motor processing, and visuospatial orientation. In this review, we evaluate the growing literature on the applications of nTMS in the preoperative setting. First, we analyze the evidence in support of the most common clinical applications. Then we identify usages that show promise but require further validation. We also discuss developing nTMS techniques that are still in the experimental stage, such as the use of nTMS to enhance postoperative recovery. Finally, we highlight practical considerations when utilizing nTMS and, importantly, its safety profile in neurosurgical patients. In so doing, we aim to provide a comprehensive review of the role of nTMS in the neurosurgical management of a patient with a brain tumor.

## Introduction

A primary tenet of neurosurgical oncology is to achieve maximal resection of pathologic lesions while preserving the surrounding eloquent brain and, thus, protecting a patient's functional ability. However, as we have continued to expand our knowledge of cognitive neuroscience and higher-order brain function, traditional theories regarding discrete brain regions housing critical functions and the general functional topography of the brain have been challenged (1–3). This anatomical description of functional brain regions is further complicated in the setting of architecture-distorting lesions (4), highlighting the necessity of additional modalities for determining eloquent vs. non-eloquent brain. One such modality is navigated transcranial magnetic stimulation (nTMS). nTMS involves the use of non-invasive, image-guided stimulation of the brain to generate a functional map that differentiates eloquent from non-eloquent tissue (5). Transcranial magnetic stimulation is accomplished by using a wound copper coil (typically in a figure-of-eight configuration) to generate a strong, magnetic pulse targeted at an area of interest. By integrating the coil with a frameless stereotactic image guidance navigation system, one can achieve highly accurate maps that are specific to each subject's unique anatomy. Frequently performed in the preoperative setting, information learned from nTMS can aid with operative planning and allow for more accurate patient risk-stratification and counseling. In this review, we discuss various uses for preoperative nTMS, such as motor and language mapping, considerations surrounding patient safety, and future directions of the field.

## Motor Mapping with nTMS

The most well-established role for pre-surgical nTMS is mapping the spatial location of functional motor areas relative to the location of the tumor (6–8). The modality has a high degree of accuracy in the preoperative identification of eloquent motor cortex, with nTMS correctly identifying the primary motor cortex in 99.7% of cases (9).

### Comparison With Direct Cortical Stimulation

Systematic comparisons between nTMS and direct cortical stimulation (DCS), the gold-standard technique for motor mapping, have demonstrated excellent concordance between the two modalities. Tarapore et al. found that the distance between TMS and DCS motor sites was ~2.1 mm (10), and other groups have replicated this high degree of spatial reliability and consistency between DCS and nTMS (11, 12). Importantly, over multiple studies, there were no positive motor mapping sites identified with DCS that were unrecognized with TMS, demonstrating the high degree of sensitivity for preoperative nTMS. Conversely, sites that were deemed non-eloquent with DCS were also found to be quiet with nTMS, indicating a high degree of specificity for nTMS vs. DCS as well. As a result, nTMS based motor maps may be thought of as interchangeable with DCS based motor maps, and both positive and negative maps may be used to guide clinical care.

nTMS based motor maps have a high degree of consistency over time and between different examiners (13–15), demonstrating excellent inter-operator reliability. Moreover, the reference range of normal values for nTMS-based motor evoked potentials (MEPs) is not affected by tumor size, location, or patient clinical/socioeconomic status, which eases the interpretation of the results (16). Additionally, mapping with nTMS has been shown to be safe to perform in patients with brain tumors, although typically it is not performed in patients who are experiencing frequent seizures, with a transient headache being the most common complication reported (17). Thus, nTMS for motor mapping a straightforward technique to add to an existing workflow for neuro-oncology patients.

### nTMS-Based Motor Maps in Clinical Practice

The high reliability of nTMS-based motor maps has enabled clinicians to improve the clinical management of patients with potentially eloquent brain tumors. Frey et al. found that nTMS disproved suspected involvement of the primary motor cortex by the tumor in ~1/4 of cases, frequently altering the surgical plan and preoperative patient counseling (17). Planned surgical resection was expanded in over 1/3 of cases and the percentage of tumors where a gross total resection was achieved increased by nearly 20%. Importantly, there was a corresponding decline in the rate of postoperative deficits in the group of patients who underwent nTMS. These findings suggest that the addition of preoperative nTMS mapping data to a clinical routine of preoperative fiber tractography, intraoperative neuronavigation, and intraoperative mapping/electrophysiology improves surgical outcomes for tumors in or near the motor pathways (18).

### Fiber Tracking With nTMS Motor Maps

In patients with glioma, MR signal alterations caused by vascular changes and peritumoral edema can create spurious DTI results and reduce the accuracy of the tractography. To improve accuracy, nTMS hot spots in the primary motor area can be used in conjunction with carefully selected subcortical nuclei seed voxels to improve the anatomic accuracy of the tracts. This technique is useful in patients whose tracts are closest to the tumor (19), as these patients are at highest risk of developing postoperative motor deficits due to intraoperative injury to the subcortical white matter (20–22). In addition to displaying highly accurate fiber tractography (FT) that can be used to plan the approach to surgical resection, it can also inform the surgeon when to employ intraoperative DCS. Furthermore, these nTMS-based DTI FT can be used in a predictive manner as well: patients with nTMS-generated CST fibers with lower fractional anisotropy (FA) values and higher ADC values are much more likely to have their motor function deteriorate postoperatively (23). In fact, nTMS localizer data produces better DTI corticospinal tractography results than functional MRI for patients with tumors near the cortical tract origin (i.e., primary motor cortex) (24).

Accordingly, Raffa et al. showed that nTMS-based CST mapping allowed for patients to receive smaller craniotomies (25). Phase reversal was rarely needed as the cortical nTMS information facilitated identification of the primary motor cortex (12), which likely allowed the surgeons to perform smaller craniotomies and decrease surgical time. Moreover, these patients had fewer postoperative seizures, improved EOR, and better postoperative KPS and motor performance after surgery, indicating the powerful benefit of tailoring the surgical approach with nTMS-based FT for motor-eloquent lesions. The authors demonstrate how nTMS-based tractography provides visual feedback that can guide ongoing resection of tumor in safe areas even when the DCS threshold is very low and would otherwise mandate the surgeon stop the resection. Interestingly, nTMS-based FT appears to be more useful in high-grade tumors, as they typically displaced the tracts without infiltrating it, further highlighting the predictive nature of nTMS-based DTI for the resectability for lesions involving the CST.

Additionally, the distance from a lesion to a fiber tract defined by pre-surgical nTMS is strongly correlated with the likelihood of developing a postoperative deficit, although the proximity threshold for when a postoperative deficit is encountered may vary depending on the specific tract (e.g., corticospinal tract vs. arcuate fasciculus) in question (26). Specifically for the corticospinal tract, lesions > 8 mm from the tract have been considered low risk in some series and no new postoperative motor deficits were observed following gross total resection of the tumor (27).

### Preoperative Risk Stratification

nTMS-based motor thresholds have also proven useful in pre-surgical risk stratification. Rosenstock et al. utilized a logistic regression model to identify preoperative nTMS-related variables that were associated with postoperative motor outcome. They found that three criteria were significantly associated with new postoperative deficit: tumorous infiltration of the motor cortex and/or CST; ≤8-mm distance between tumor and CST; interhemispheric resting motor threshold <90% or >110%. Of note, patients with a pre-existing motor deficit and impaired cortical excitability in the tumorous hemisphere on nTMS never showed a postoperative improvement in motor function. However, patients with equally excitable hemispheres (similar to healthy subjects) have better outcomes and may be considered lower risk (27). These findings highlight the important role of nTMS-based resting motor thresholds in measuring comparative cortical excitability. Not only does this risk-stratification strategy allow for improved surgical planning, it also improves the specificity of patient counseling with regard to perioperative risk. Accordingly, the planned extent of resection has been shown to change often in patients who undergo preoperative nTMS, with an increase in surgical aggressiveness being the most common conversion made to the surgical plan after nTMS assessment (17).

### Improvement in Outcomes

Accordingly, preoperative nTMS has consistently been shown to facilitate more extensive resections while reducing functional deficits, and thus improved patient survival (6, 12, 17, 28).

Frey et al. showed that the rate of gross total resection (GTR) in patients who underwent preoperative nTMS was significantly increased compared to a control group of patients who did not undergo preoperative nTMS (17). The higher proportion of patients receiving a GTR resulted in a 7-month prolongation of progression free survival for the low-grade glioma nTMS cohort relative to the non-nTMS cohort (22.4 vs. 15.4 months, respectively). As mentioned above, the nTMS cohort also had a small drop in the rate of postoperative deficits.

**Figure 1:**
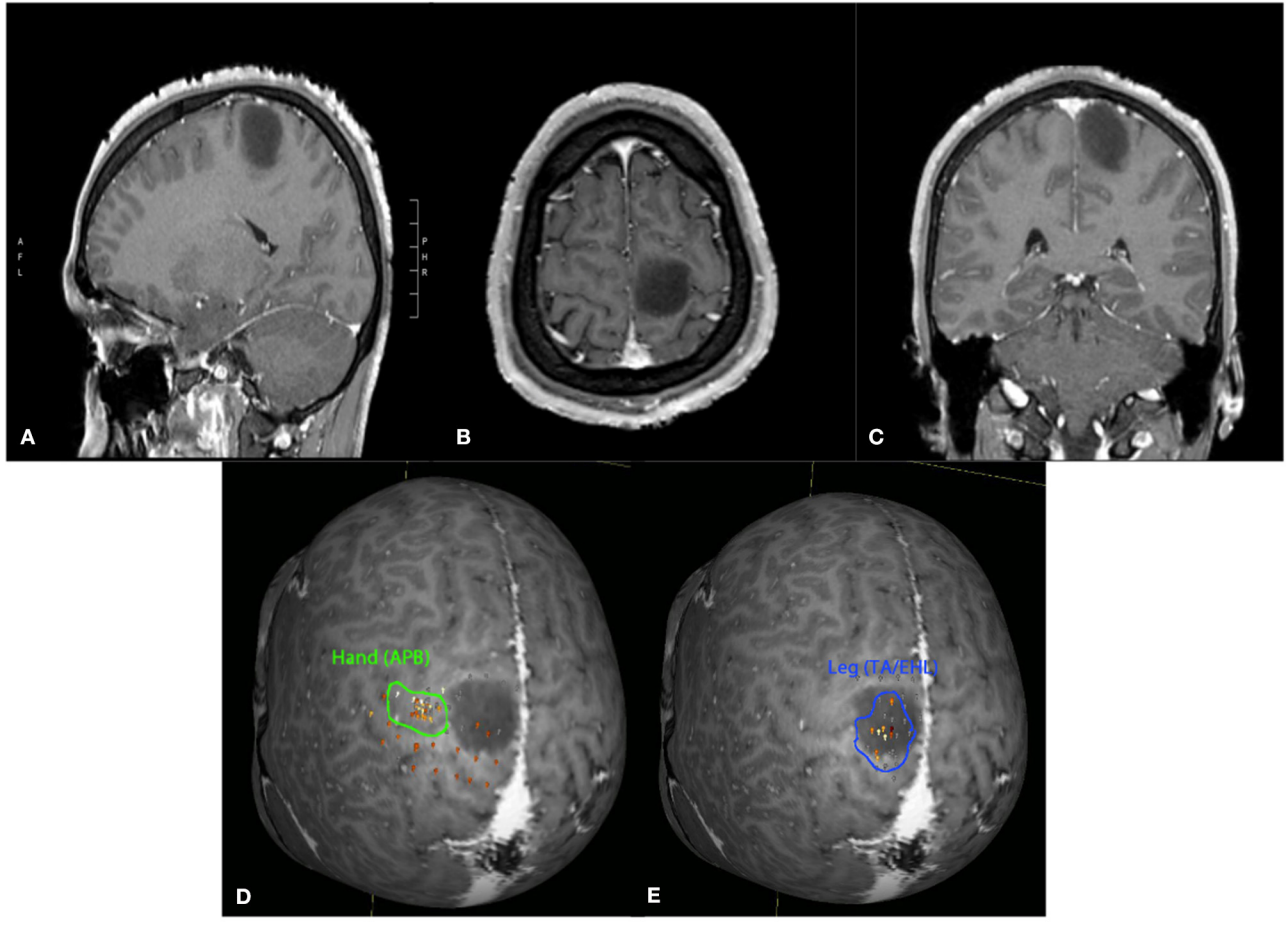
Twenty-four-year-old male with an incidentally discovered brain lesion, presumed low grade glioma, within the left paracentral lobule (T1 post-contrast imaging, **A–C**). nTMS demonstrated hand motor responses lateral to region of the lesion, but leg responses were present throughout the lesion **(D,E)**. Surgery was deferred due to the nTMS mapping results and increased risk of potential lower extremity motor deficit following surgical intervention.

**Figure 2:**
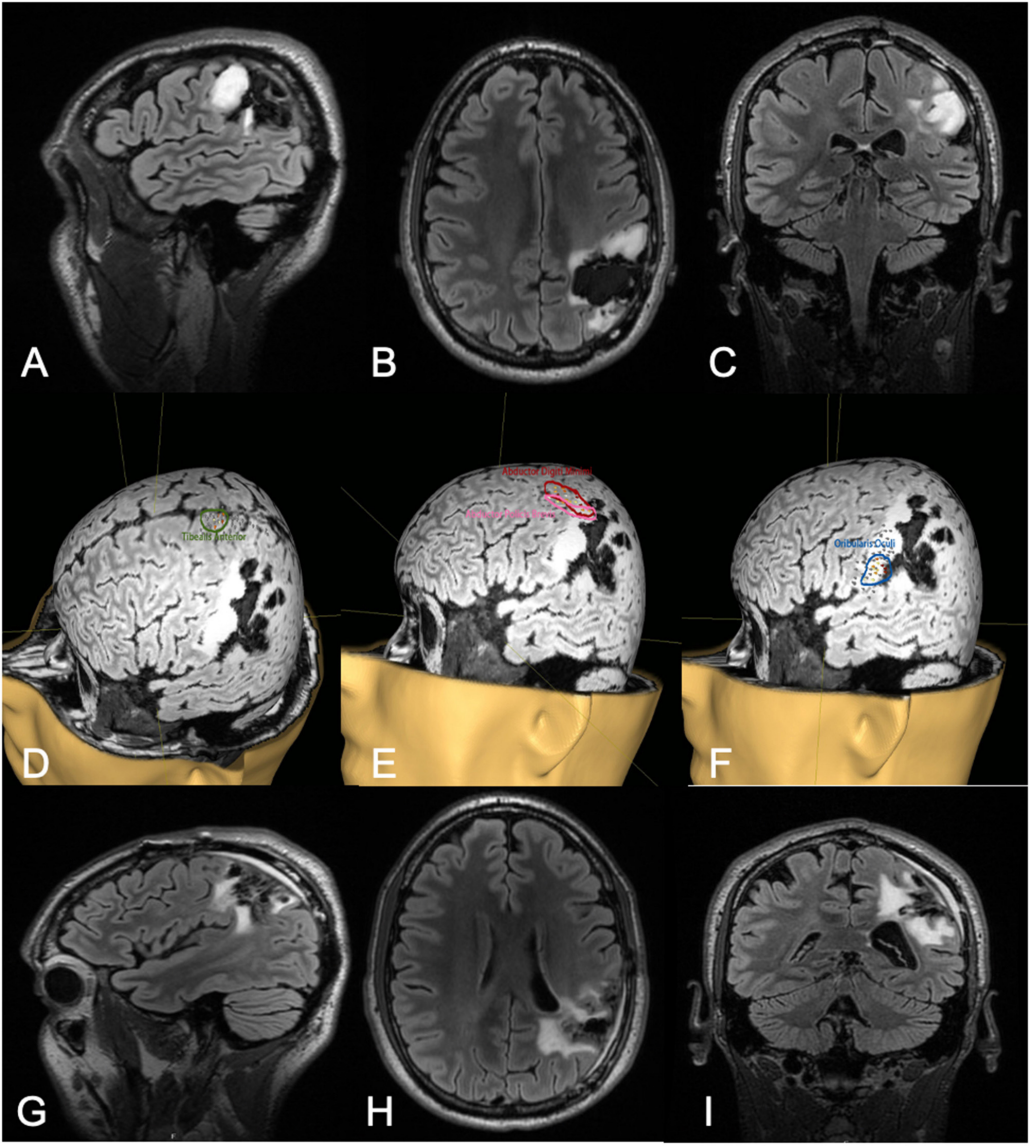
Thirty-two-year-old male with a history of left parietal oligoastrocytoma status post prior resection, now presenting with recurrence (T2 FLAIR imaging, **A–C**). nTMS demonstrated hand motor responses anterior to the region of recurrence, face motor responses entirely within the recurrence, and leg motor responses anterior to the recurrence **(D–F)**. Intraoperative DCS identified hand and upper extremity function in close proximity to, and at times continuous with, the area of recurrence. Subtotal resection was achieved with care taken to spare hand and face motor sites intraoperatively (T2 FLAIR imaging, **G–I**). The patient had no postoperative neurological deficits on neurological examination.

**Figure 3:**
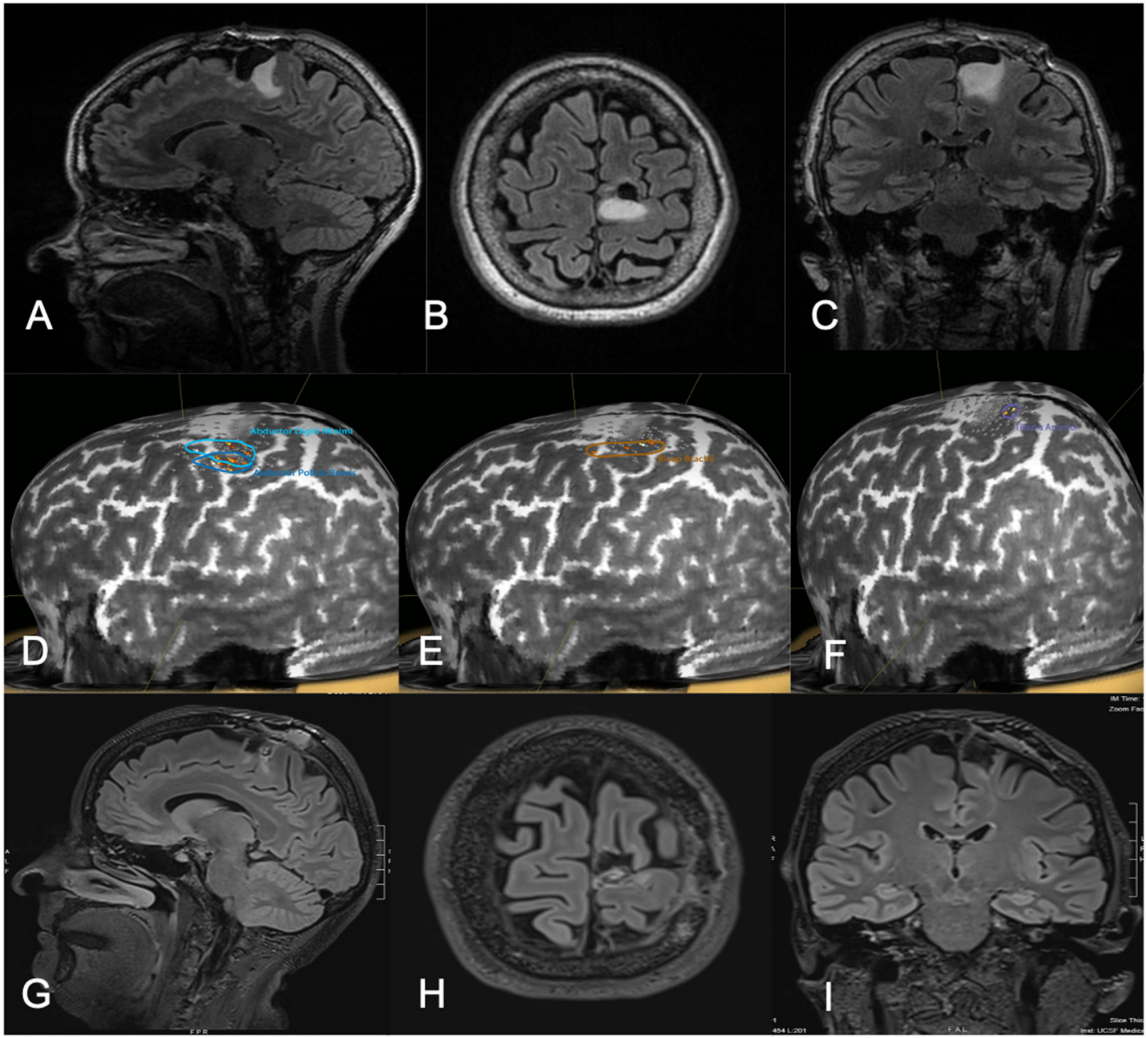
Fifty-one-year-old female with a history of left parietal oligodendroglioma status post prior subtotal resection, now presenting with recurrence (T2 FLAIR imaging, **A–C**). nTMS demonstrated hand and lower extremity motor activity lateral and posterior to the area of prior resection and region of recurrence **(D–F)**. Intraoperative DCS identified lower extremity motor function in close proximity to the area of recurrence, as identified on preoperative nTMS. Subtotal resection was achieved with care taken to spare motor sites intraoperatively (T2 FLAIR imaging, **G–I**). The patient had no postoperative neurological deficits on neurological examination.

Krieg et al. completed a prospective study of 100 patient with supratentorial lesions located in the motor region who underwent preoperative nTMS and compared their outcomes to a matched control group who were operated on at the same institution at a prior time without nTMS (28). Consistent with Frey's prior report, the authors found that there was a lower rate of residual tumor on the post-operative MRI and that ~10% of patients in the nTMS group had an improvement in their motor function, which was much higher than the 1% of patients who had an improvement in motor function in the non-nTMS group. Similar to prior reports, there was a lower rate of postoperative motor decline in the nTMS group relative to the non-nTMS group (13% vs. 18%). Finally, a systematic review and meta-analysis of preoperative nTMS motor mapping by Raffa et al. demonstrated a significant reduction in new permanent postoperative motor deficits and increased GTR in patients who underwent preoperative motor mapping relative to those who did not. Although these reports are not randomized control trials, the evidence supports incorporation of nTMS into the work-flow and imaging arsenal for patients with eloquent tumors in the motor region and should be used in combination with intraoperative mapping to optimize patient outcomes. Case examples highlighting the benefit of preoperative motor mapping using nTMS can be found in Figures 1–3.

### Prognostic Value in Recovery

nTMS motor mapping has shown promise in predicting recovery for patients with new postoperative deficits. In the first study utilizing pre- and post-operative nTMS for prognostication of recovery potential, Takakura et al. showed that when preoperative cortical hotspots (defined as cortical regions that elicited the largest EMG in the adductor hallucis brevis by nTMS) are adjacent or within 1 cm to a postoperative lesion, there is less recovery of hand grip strength compared to patients whose cortical hotspots were more distant from the postoperative lesion (29). The group with lesions adjacent to the pre-surgical nTMS hotspots had only recovered by 55% 3 months after surgery compared to patients with non-adjacent lesions who had recovered by 95% during this time. This finding is particularly important given the fact that nearly all patients will have a decline in their motor function immediately following surgery due to postoperative edema, highlighting the utility in nTMS to predict which patients are most likely to recover and the degree of that recovery. Equally importantly, there was no correlation between an intraoperative decline in MEP signaling and postoperative grip strength or recovery, highlighting the unsuitable nature of MEPs for predicting recovery from postoperative motor deficits. Finally, positive postoperative nTMS-MEPs 1 week after surgery correlated well with better recovery from an immediate postoperative deficit, which corresponds well to the post-stroke literature and represents one of the few prognostic tools that can be used to evaluate patients with new motor deficits after glioma surgery.

## Language Mapping

While presurgical motor mapping remains the most common and well-validated application of nTMS, language mapping is also an exciting area of potential clinical utility for nTMS and has been under investigation since the early 1990s (30). In contrast to motor mapping, which uses single pulses to excite neurons and cause downstream motor function, language mapping uses short bursts of TMS pulses, called repetitive TMS or rTMS, to cause a temporary lesion and disrupt the normal function of the brain. When these pulses are guided by neuro-navigation, they are referred to as navigated repetitive TMS (nrTMS). Although the mechanism of action is not entirely understood, synchronization of affected neurons and GABAergic inhibition are thought to contribute to the temporary brain disruption and lesion effect (31). Because of its non-invasive, reversible effect, nrTMS provides a valuable modality with which to map eloquent language regions of the cortex. Preoperative language mapping is especially valuable in brain tumor patients as, due to tumor induced plasticity and remodeling, eloquent language areas may be shifted to unexpected cortical regions (32, 33).

### Initial Studies With rTMS

Early studies investigating the use of TMS in language mapping focused on determining hemispheric language dominance and utilized rTMS without navigation. Pascual-Leone et al. first highlighted the ability of rTMS to induce speech arrest in a study involving six epileptic patients, demonstrating identical lateralization results to intracarotid amobarbital tests performed on the same patient and hinting at the potential clinical utility of the technology (30). This experiment was replicated by Jennum et al. (34). However, a subsequent study by Epstein et al. in 2000 (35), highlighting inconsistencies between rTMS and intracarotid amobarbital tests, showed that the development of postoperative deficits were more effectively predicted by an intracarotid amobarbital test. This result cast some doubt on the utility of rTMS in determining hemispheric language dominance (9, 35, 36). A number of similar studies investigating rTMS alone provided unreliable results secondary to inconsistencies with intracarotid amobarbital tests, more specifically high false-positive speech arrest sites in the non-dominant hemisphere (36, 37). Studies also failed to correlate rTMS language mapping findings with DCS. These results highlighted the need for improved targeting, and controlling more specifically the perturbations of the functional landscape, and was a primary driver for integrating the rTMS system with neuro-navigation. This effort, it was hoped, would permit more detailed investigations into the relationship between TMS findings and intraoperative DCS.

### Initial Language Studies With nrTMS

Fortunately, the incorporation of neuro-navigation into rTMS improved upon the results seen in previous studies using rTMS alone. Tarapore et al. sought to demonstrate the utility of nrTMS through a study of 12 brain tumor patients who also received intraoperative DCS. Using intraoperative DCS as the gold-standard test, they demonstrated that nrTMS had a sensitivity of 90% and a specificity of 98% for detecting speech-language disruption sites (positive predictive value of 69% and negative predictive value of 99%), highlighting the accuracy and utility of nrTMS (37). However, the predictive values reported by Tarapore et al. were significantly higher than a similar study performed by Picht et al. in 20 patients undergoing awake resection of a brain tumor. Also using DCS as a gold-standard test, they reported significantly lower predictive values: sensitivity of 90.2%, specificity of 23.8%, positive predictive value of 35.6%, and negative predictive value of 83.9% (38). Given the similarity in methodology between the two studies, a difference in the definition of a language disruption site is thought to contribute to the discordance in predictive values observed (9). Indeed, Tarapore et al. which demonstrated a higher correlation between nrTMS and DCS, utilized a slightly more stringent definition of language disruption, requiring the agreement of two blinded experts with the utilization of a third expert in the case of a disagreement (9, 37). A subsequent study by Raffa et al. took a slightly different approach, using postoperative aphasia (determined by the Western Aphasia Battery) as a gold-standard measure to determine the accuracy of preoperative nrTMS and nrTMS-based DTI FT in brain tumor patients unable to undergo intraoperative DCS. They also demonstrated a good correlation between preoperative nrTMS and development of a postoperative deficit: sensitivity 100%, specificity 57.14%, negative predictive value 100%, positive predictive value 50%, further highlighting the promise of this technology (33). Indeed, the high negative predictive value seen by Raffa et al. especially demonstrates the value and reliability of a negative test result, which can significantly aid with preoperative planning and identifying non-eloquent regions of the brain. In the largest study to date, Sollmann et al. utilized data from 100 patients undergoing preoperative language mapping. Using deterministic tractography based on nrTMS data, they demonstrated a significant relationship between lesion to tract distance (LTD) and the development of permanent post-surgical language deficits with cutoffs of ≤16 mm LTD for the arcuate fasciculus and ≤25 mm LTD for other closest language-related tract (26). This study again highlighted the utility of nrTMS, more specifically LTD, as a preoperative risk stratification tool.

### The Role of nrTMS in Multi-Modal Presurgical Language Mapping

Thus, while nrTMS is an improving technology for preoperative language mapping, it remains less accurate than when used for motor mapping, as previously discussed. This has prompted the combination of nrTMS with other non-invasive technologies, such as functional MRI (fMRI) leading to improved predictive ability of language disruption sites when the two are used together (39, 40). Additionally, a growing literature has described the benefit of seeding tractography maps with nrTMS-based language disruption sites. This is especially critical in cases requiring more accurate subcortical language mapping. Sollmann et al. demonstrated the feasibility nTMS based DTI FT of subcortical language pathways in 2016, highlighting the ability of the two technologies together to identify nine language-related subcortical tracts (41). Raffa et al. subsequently showed that nTMS combined with DTI FT allowed for a more accurate and reliable reconstruction of the subcortical language network when compared to standard DTI FT using anatomical landmarks, further demonstrating the synergistic nature of the two technologies (42). Interestingly, Sollmann et al. then demonstrated the ability to produce a function specific DTI FT when only specific language errors following nrTMS were utilized as regions of interest for DTI FT; highlighting the ability to more specifically map subcortical functions (43). In a separate study, Sollman et al. described the clinical use of nrTMS and nrTMS-based DTI FT. While the study described some clinical outcomes, including craniotomy size, extent of resection, and postoperative language deficits it lacked a control group, making it difficult to appreciate the full impact of these technologies a patient's outcome (44). Nevertheless, it was a first step toward much needed studies, such as a randomized controlled trial, in which any potential clinical benefits associated with nrTMS-based DTI FT could be more clearly described. Finally, nrTMS-based DTI FT has also demonstrated use in preoperative risk stratification of patients with tumors in language eloquent regions. Sollman et al. sought to define the LTDs on nrTMS-based DTI FT that predicted postoperative surgical deficits in 50 patients with left hemispheric language eloquent brain tumors. They demonstrated LTDs of ≤8 mm for the arcuate fasciculus and ≤11 mm other language-related tracts as cutoffs for surgery-related permanent aphasias (45). Of note, these cutoffs were closer than those determined in a similar study using deterministic tractography based on nrTMS, highlighting the promise of nrTMS-based DTI FT (26).

Consideration should also be given to other patient characteristics and variables that could contribute to the reduced predictive value of nrTMS, including pre-existing aphasia or cognitive deficits (46). Mitigation strategies and modified protocols that increase the utility of nrTMS for language mapping in these patient populations should continue to be explored. Given the complexity and variability of language function, improved nrTMS language mapping protocols are needed and will continue to be developed. Nevertheless, nrTMS for language mapping remains an exciting technology with the ability to positively impact patient care in the clinical setting. This is especially true for patients who cannot tolerate awake intraoperative language mapping, as nrTMS provides surgeons with a way to improve safety and increase eligibility for surgery in patients who might otherwise be deemed inoperable (33).

## Future Directions

Despite a historical focus on motor and language mapping (47), it is also clear that additional brain functions contribute significantly to patient quality of life following surgery, ranging from vision to complex higher level cognitive functions. Indeed, a number of functions, including vision, spatial awareness, memory, attention, judgement, emotion, and calculation have been mapped intraoperatively (48). However, adding complex tasks to evaluate these cognitive functions can add a large amount of time during the awake, intraoperative mapping portion of a case which can be challenging for patients to tolerate and increase the duration of the surgical procedure, making it difficult to use these on a regular basis. Thus, the use of nTMS for the preoperative mapping of complex functions is an attractive option as this preoperative mapping occurs in a setting where more time can be taken to dissect these intricate relationships.

### Visual Cortex With nTMS

For example, one of the first regions outside of language and motor mapping to be mapped using nTMS was the visual cortex (49). In 2002, Fernandez et al. demonstrated the ability for TMS to systematically map visual sensations; they consistently evoked reproducible topographically organized phosphenes (a brief flash of light) through the use of TMS, demonstrating the reliability and reproducibility of the technology. Subsequent studies have shown that a weak TMS pulse to the visual cortex will often result in the patient seeing a phosphene while stronger pulses tend to have a more suppressive impact on the visual cortex (50). Salminen-Vaparanta et al. also demonstrated the ability to selectively stimulate the primary visual cortex when using TMS in conjunction with multifocal functional magnetic resonance imaging (mffMRI) to first identify individual retinotopic areas. However, even when using mffMRI data, the primary visual cortex was only able to be stimulated in half of the tested patients, highlighting the inaccuracies and difficulties associated TMS stimulation of the visual cortex, even when image-guided (50). Thus, while mapping of the visual cortex with TMS remains an exciting possibility, additional research is needed to refine and develop this application.

### Experimental Mapping Techniques With nTMS

TMS has also been used to investigate complex functions, such as visuospatial attention and spatial orientation (51–53), facial recognition (54), and calculation (55, 56). With regards to visuospatial perception, Salatino et al. utilized a line length estimation task to capture the development of neglect when stimulating the right posterior parietal cortex (PPC) with single-pulse TMS. Interestingly, they demonstrated the development of left sided neglect when stimulating over both the right and left PPC. However, in a follow-up study, they demonstrated neglect when performing rTMS on the right PPC, but not the left, highlighting the ability of TMS to cause neglect while also suggesting that rTMS, rather than single-pulse TMS, may be a more accurate way to map visuospatial perception (52, 53). rTMS has also shown use in identifying cortical regions associated with visuospatial attention, another exciting potential future application of this technology (51).

Given the ability for brain tumor resections, especially in the frontal and parietal lobes, to cause prosopagnosia, efforts have also been made to map other complex functions, such as facial recognition. Maurer et al. sought to explore the mapping of facial recognition function in 20 volunteer patients by targeting 52 regions of the cortex with nrTMS and simultaneously testing the ability to name popular celebrities. They identified a number of locations that lead to naming dysfunction when nrTMS was applied to them; 80% of all participants demonstrated a naming error when nrTMS was utilized over the middle frontal gyrus (54). This study demonstrated the feasibility of utilizing nrTMS for mapping of facial recognition. Future investigations will likely evaluate this application of nrTMS in the setting of preoperative planning.

Finally, a number of studies have investigated the use of nrTMS to map the ability to perform mathematical calculations. Similar to how they tested the mapping of facial recognition, Maurer et al. also mapped calculation function by asking patients to perform simple arithmetic tasks while applying nrTMS to 52 predetermined cortical locations. Interestingly, an 80% error rate was observed when nrTMS was applied to the right ventral precentral gyrus, with different types of arithmetic localizing to different regions of the brain (e.g., division tasks showed the highest error rate in the left middle frontal gyrus) (55). Similar findings, with the segregation of specific types of arithmetic, were demonstrated by Montefinese, further highlighting the potential utility of nrTMS for mapping calculation ability.

While promising, the clinical utility and applicability of the aforementioned experimental mapping techniques still need to be investigated and validated. This will likely be accomplished through studies similar to what have already been performed for language and motor mapping, in which the predictive value of preoperative nTMS is assessed by comparing it to intraoperative DCS or postoperative deficits. Until then, assumptions regarding the clinical utility of these experimental mapping techniques in the setting of preoperative mapping should be considered with caution.

### Postoperative Therapeutic Applications of nTMS

Finally, it is worth briefly discussing the use of TMS as a therapeutic intervention for patients with stroke, traumatic brain injury, or postoperative injuries. In the injured brain, rTMS is thought to have a beneficial effect by potentially reducing cortical hyper excitability and promoting long-term plasticity (57). Preliminary studies into the potential therapeutic benefit of TMS or transcranial direct current stimulation in patients with a stroke or brain injury have been promising, with studies highlighting the potential for these technologies to improve motor function in stoke patients (58, 59) as well as improve working memory (60, 61). While a more recent study has showed no beneficial impact of rTMS on cognition in TBI patients (62), additional investigation into the use of this technology as an adjunct to aid in recovery following an injury to the brain is warranted and holds promise for an expanded role for TMS in the management of patients afflicted by these conditions.

## Safety

TMS is traditionally viewed as a safe technology, when the appropriate stimulation parameters are followed (63). The most common side effects associated with use of the technology are minor and include: pain (39–40%) (64, 65), headache (28–40%) (64, 65), and high frequency hearing loss (9%) (30, 66). While minor side effects are tolerable for the majority of patients, the most severe, and feared, complication of TMS is the development of a seizure. Fortunately, rates previously reported in the literature have indicated a <1% incidence of seizure related to TMS (67–70). Despite the low incidence of TMS associated seizure, the United States Food and Drug Administration requires the exclusion of patients with poorly controlled seizures (>1 seizure per week) when using TMS for preoperative mapping. However, the paucity of data surrounding TMS side effects in preoperative neurosurgical patients specifically prompted a large study by Tarapore et al. investigating the safety profile of TMS in 733 preoperative neurosurgical patients, 50% of which had preoperative seizures related to their lesions. Interestingly, while some discomfort or mild to moderate pain was reported by patients, especially those receiving rTMS, no TMS associated seizures or persistent headaches were reported; this highlighted the strong safety profile of TMS and the potential of the technology to be used in all patients, including those with a history of seizures.

## Conclusion

**Figure 4:**
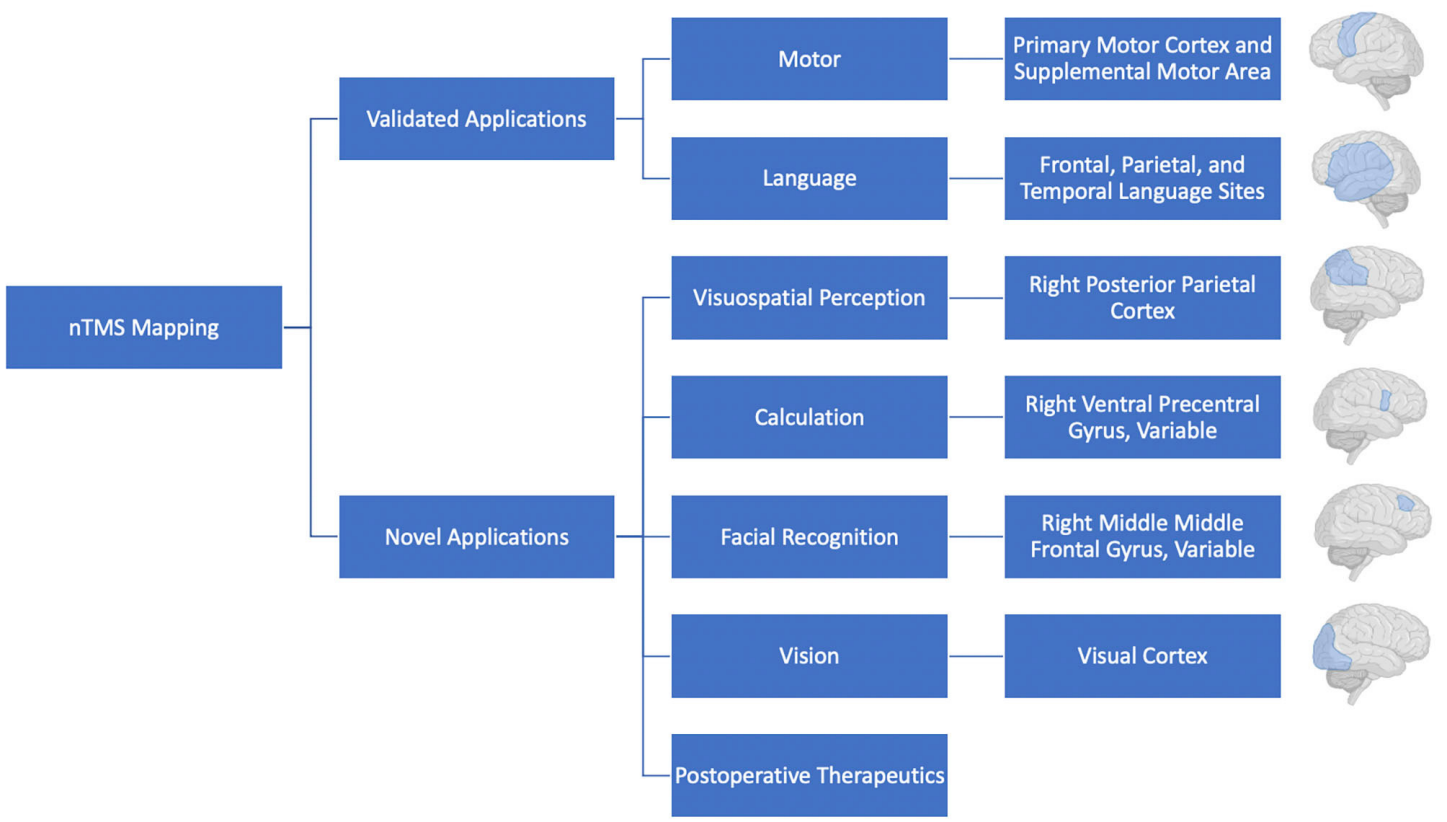
Schematic highlighting common uses of nTMS.

TMS will undoubtedly continue to see use for preoperative motor and language mapping, with additional mapping of complex functions likely to become more common as they are validated in the clinical setting (Figure 4). The combination of nTMS with additional mapping modalities, such as fMRI also holds great promise and should also continue to be explored. As we continue to learn more about and refresh our view on the functional topography of the brain it will be increasingly important to provide surgeons with more accurate, personalized, representations of a patient's brain. As a result, preoperative mapping using TMS has the potential to contribute to operative planning, improved patient risk-stratification, and better-informed patient counseling. It is an exciting technology that will continue to see investment and use in the neurosurgical and neuroscience communities.

## Author Contributions

All authors listed have made a substantial, direct and intellectual contribution to the work, and approved it for publication.

## Conflict of Interest

The authors declare that the research was conducted in the absence of any commercial or financial relationships that could be construed as a potential conflict of interest.
